# Multi-Morbidity in the Older Person: An Examination of Polypharmacy and Socioeconomic Status

**DOI:** 10.3389/fpubh.2020.582234

**Published:** 2021-01-18

**Authors:** Martin C. Nwadiugwu

**Affiliations:** ^1^Faculty of Health and Sports University of Stirling, Stirling, United Kingdom; ^2^Department of Biomedical Informatics, University of Nebraska, Omaha, NE, United States

**Keywords:** polypharmacy, multi-morbidity, socioeconomic status, older adult, person-centred care, care approach

## Abstract

There has been increased focus on clinically managing multi-morbidity in the older population, but it can be challenging to find appropriate paradigm that addresses the socio-economic burden and risk for polypharmacy. The Commission on Social Determinants of Health (CSDH) has examined the need for institutional change and the parallel need to address the social causes of poor health. This study explored three potential interventions namely, meaningful information from electronic health records (EHR), social prescribing, and redistributive welfare policies from a person-centered perspective using the CARE (connecting, assessing, responding, and empowering) approach. Economic instruments that immediately redistribute state welfare and reduce income disparity such as direct taxation and conditional cash transfers could be adopted to enable older people with long-term conditions have access to healthcare services. Decreased socioeconomic inequality and unorthodox prescriptive interventions that reduce polypharmacy could mitigate barriers to effectively manage the complexities of multi-morbidity.

## Introduction

The term multi-morbidity describes the presence of more than two long-term conditions within an individual (1, 2). Although the term is used inconsistently and interchangeably with co-morbidity, it is different from co-morbidity, which is the concurrent presence of two long-term conditions that may occur before or after the onset of a disease of interest (3). Multi-morbidity is a complicated and socially patterned phenomenon associated with socio-economic deprivation (1), because people with multi-morbidity usually face social, mental, and physical health conditions that require care from multiple services, and are often in need of support for additional unplanned emergency care, which makes coordinating their care challenging (2, 4). While some people with complex conditions might require a tailored approach such as implementing an individualized management plan, reviewing medicines, establishing treatment burden, and other recommendations in the NICE guidelines, this may not be beneficial for others with related and well-controlled long-term conditions (4). A lack of understanding of the complexities of multi-morbidity, which is often impacted by social factors that affect treatment and improved patient experience, complicates healthcare interventions for people with the condition (5, 6).

It is estimated that one in three adults globally has multiple morbid conditions (7). In Scotland for example, it was reported that older people (>65+ years) are living with three or more long-term conditions, and predictions indicate that in 20 years there will be an increase of ~50%, which will invariably lead to increased demand for social and health care services (8). This implies that more funding for social and health care services will be needed to respond to trends in the population demographic profile due to an increase in older populations and socioeconomic inequality (8, 9).

Socio-economic inequality is a challenge that complicates the management of multi-morbidity among older people due to the high cost of health care, and the disparity in income distribution for less affluent older people in both developed and developing countries (10). In the US, for example, the poverty rate among older people aged 65 and over is 9.7% (11), and about 7 million older adults are living below the national poverty line (10). Economically, poor older populations experience unequal access to health care due to their social-economic position. Apart from the socio-economic problems faced by less affluent older people with multi-morbidity, healthcare providers spend huge sums of money annually managing patients with multi-morbidity (12) compared to those without a long-term condition (13). For example, in Singapore, the annual economic cost of managing an additional long-term condition is increased by SGD$3,177 and SGD$2,265 for social and health care respectively (14). This continuous spending on patients with multi-morbidity is unsustainable (15); and there is a need for a more holistic approach to the complexities of multi-morbidity (16). Scientific attention has been drawn to the fact that, apart from the differences in health conditions between the affluent and the less affluent in society, the social determinants of health (sensitivity of one's health to the social environment) is an important area for understanding the complexities of multi-morbidities (16).

According to the WHO (16), socioeconomic factors are a determinant of good health throughout life. Older people who are economically poor and isolated are more likely to have long-term diseases and higher levels of disability, especially when they do not receive social care that meet their needs (17). This is especially true because socioeconomic inequality could lead to social isolation (18) and as social beings that live in a society, we need friends, social connections, and meaningful work, to feel appreciated and valued. Without socioeconomic protection, older individuals become susceptible to depression and other physical and mental health conditions (16) and the severity of these conditions are also connected to social status (19).

The challenges around clinical management models, socio-economic status, and the healthcare burden arising from multi-morbidity and increased risk of adverse drug reactions (ADR) are continually emerging topics of discussion among healthcare providers and policymakers (5). The onset of multi-morbidity is present in about 98% of older population (20), who often need treatment with multiple medications specific to the disease, pre-disposing them to the risk of polypharmacy, which is the use of medications not clinically indicated. This further increases the complexity of managing multi-morbidity for both physicians and patients (1, 21, 22).

This paper studies the complexity of multi-morbidity among older people (≥65 years) in relation to socio-economic status and polypharmacy. It examines the influence of socio-economic inequalities, drawing from the Commission on Social Determinants of Health (CSDH), a global network brought together by the WHO that aims to address the social causes of poor health and health inequity by inspiring policy and institutional change (23). The CSDH framework shows how socio-economic and political mechanisms such as income, housing, education, and employment gives rise to economically stratified populations, which in turn, shapes health determinant status and differences in vulnerability to morbid conditions (24, 25). These factors play a role in shaping the lives of older people and can determine the extent to which the complexities of multi-morbidity are managed (25). The study proposes implementing redistributive welfare policies or social program that reallocate wealth to citizens in order to reduce socioeconomic inequalities. The use of information from electronic health records and social prescribing to prevent unnecessary drug prescription were two other suggested interventions discussed using the CARE approach (26, 27). The CARE approach is an important framework for person-centered and empathic encounters in healthcare relationships. It is used to understand a patient's situation, perspective, and underlying meanings, and to communicate this accurately in a flexible and helpful way (27). It involves actively engaging with a patient to open communication lines (connecting), listening for attached meanings (accessing), accurately communicating this understanding (responding), and planning measures in partnership with them (empowering) (27). The flexibility offered by this approach allows for different guiding principles to be applied to different circumstances.

This paper will be organized first by highlighting the complexities of multi-morbidity in relation to polypharmacy and socio-economic status. Next, person-centered interventions namely, social-prescribing, electronic health records, and models for effective communication between patients and healthcare providers will be presented using the CARE approach. The study will discuss ways society can redistribute welfare so that income disparity is reduced, and will conclude with a summary of how older people may benefit from the interventions.

## The Complexities of Multi-morbidity in the Older Person

Most people above 65 years of age live with multi-morbid conditions (28), and no established index exists for measuring the complexity of multi-morbidity based on socio-economic indicators (29). Some studies have attempted to develop instruments to measure the condition (30) by reviewing measures used in primary and community care settings. As Huntley et al. (31) reiterated, the lack of standardized indicators for accessing and measuring multi-morbidity are due to its complex nature.

Multi-morbidity among older people is associated with the risk of ADR arising from multiple drug combinations which are unnecessary and preventable, and from drug-disease interactions (32, 33). Budnitz et al. (34) reported that ADR was common in one-third of emergency visits by older adults. This correlates with a previous report by Goldberg et al. (35) who stated that patients who took two drugs at the same time were found to have a 13% risk of ADR, while those who took four, seven, or more medications simultaneously were 38% and 82% at risk, respectively. Polypharmacy may arise from self-prescription due to lack of affordable healthcare, and from multiple treatments for poorly managed pain in older adults with long-term conditions such as musculoskeletal diseases, cancer, insomnia, and depression (36, 37). One study reported that 60–75% of older people aged 65 years have persistent pain, and 80% of this population experience pain resulting from osteoarthritis, while ~25–50% of older people experience significant levels of pain from cancer (28, 38, 39). Moreover, these older patients are usually on high doses of pain medication and frequently seek multidisciplinary pain management services that could leave them being administered multiple drugs in a bid to manage chronic pain, thus increasing the risk of polypharmacy (40).

In addition to chronic pain caused by multiple diseases, sustained lower levels of social interaction have been suggested to contribute to depression, impaired function, and pain in older people because of a lack of social support which is known to have a buffering effect on pain. It is therefore important to also understand multi-morbidity within a social context (41). Increased interaction and togetherness between an older person and family or neighbors, rather than with formal services is suggested to be an important aspect of pain management that potentially lowers the risk of polypharmacy (42). This is because the social connections one has can provide emotional support that helps reduce psychological distress experienced as a result of failing health and insufficient income.

The prevalence of multi-morbidity has been associated with inequality in income across population groups, leading to a situation where affluent groups have the socio-economic and political mechanisms to afford medical care, making their care coordination less complex compared to less affluent groups, stratified according to income, education, occupation, gender, and race (43). These health determining social factors are associated with the presence of multiple long-term conditions (44) and governed by fundamental structural mechanisms such as the presence or absence of good governance, redistributive state welfare policies or social welfare programs, and educational attainment, all of which shape social hierarchies and are some of the root causes of health inequities and exclusion that make access to health care services difficult for people with lower socioeconomic status (44, 45).

Socioeconomic inequalities are not necessarily solely related to health care systems. Although health inequalities may very well amplify social inequalities, Mackenbach (46) stated that the relationship between health inequalities and socioeconomic inequality is probably more than a consequence of social inequality, as health inequalities have persisted and widened even in welfare states with generous arrangements. While Kohler (45) highlighted a positive correlation between higher GDP and lower socio-economic inequality, more studies are still required to establish this relationship. The non-elimination of the inability to access welfare resources and homogeneity of lower socio-economic groups in association with ill-health were two of the three circumstances suggested as plausible reasons that health inequalities have persisted in states with generous welfare arrangements (47). Another report discussed how a global within-country increase in unequal income over the past three decades was due to a decline in redistributive policies (45). While income-generating assets are important in determining income inequality, the non-effective implementation of welfare redistribution leads to a suboptimal outcome (47). This clarifies the intertwined and distinct relationship between socioeconomic inequalities, health inequalities, a welfare state, and access to health care systems.

A complexity survey by primary care physicians in Scotland rated socioeconomic and behavioral factors, rather than medical diagnosis as the major driving factors of complexity for patients (48). In addition, a study by Barnett et al. (43) using clinical data from registered patients in 314 medical practices in Scotland, revealed that less affluent people living in deprived areas were more likely to be diagnosed with long-term obstructive pulmonary disease, depression, and pain disorders. This implies that multi-morbidity in older individuals is potentially worsened by one's socio-economic potential to afford treatment for current co-morbid conditions. Moreover, the expensive cost of treatment from unplanned care and medication can force people with lower socioeconomic status further into poverty (5).

Taking care of people with multi-morbid condition cost billions of dollars annually. A study estimating the economic cost of multimorbidity among older people in Singapore found that about SGD$15,148 was the total annual societal cost required to take care of an older person with multiple long-term condition as opposed to SGD$5,610 for an older person with one long-term condition and SGD$2,265 for those with no long-term condition (14). The overall population cost was estimated at SGD$4.37 billion and was driven by the cost for social care rather than healthcare costs (14). This finding implies a linear relationship between the number of long-term conditions and increased health care expenditure. It is supported by Lehnert et al. (49) and also by Asmus-Szepesi et al. (50) who have reported a mean formal and informal healthcare costs of €30k euro per older person per year in the Netherlands. The calculated informal healthcare costs between hospital discharge and a year follow-up was €9,5k (50), showing that the cost of unpaid caregiving is substantial. The same can be seen in the United States where the estimated cost of unpaid caregiving for people with dementia is between $159–215 billion (51, 52).

## How Society Can Redistribute Welfare so that Income Disparities are Reduced

Redistributive policies are a vital tool for decreasing socioeconomic inequity, however, this cannot be achieved under a model that ultimately uses private investment-led economic growth as a criterion of progress, but instead, one that acknowledges the firm placement of the economy in society to maximize human well-being (53, 54). Measures to reduce socio-economic inequalities should incorporate asset/wealth inequality and not only economic metrics to optimize inequality reduction; this involves the equitable distribution of assets between the public and private sectors (45). Addressing equitable redistribution of wealth, affordable social care and health care among older individuals will help mitigate the complexity inherent in managing multi-morbidity in the older population (23).

Although there is no easy solution to effectively reduce income disparities, shifting resources from the affluent to those at the bottom of the income scale through taxation is one way to give older people with long-term conditions access to health care, and the most direct and immediate way to reduce inequality and social tensions (55). Apart from progressive taxation, increasing opportunities for growth and cash transfers are other instruments that can be implemented to keep inequality in check (55). Cash transfer programs that give older adults with multi-morbidity access to healthcare on the condition that they adhere to medical treatment and maintain contact with healthcare services are practical approaches to sustaining welfare redistribution. Examples of conditional cash transfers include those that require regular school attendance for children in developing economies and other cash transfer initiatives such as Mexico's *Prospera* and Brazil's *Bolsa Fam*í*lia*, which have been successful (55). Conditional cash transfers in the form of micro-credits, transportation, education, and training for carers are measures that can directly impact on the quality of life for the older person, assisting them in coping with adverse shocks pertaining to their circumstance. While income taxation and redistribution is a direct way to reduce inequality, it is, however, a short term measure with less future growth; indirect taxation on the other hand may not close the inequality gap in the short term but could lead to future growth unlike subsidies on consumption which do not effectively redistribute wealth, because the affluent also enjoy these measures (55, 56).

## Intervention for Polypharmacy Using a Person-Centered Approach

Multi-morbid disease management templates that take into account factors such as polypharmacy and social exclusion are a way to connect with, access, respond, and empower older people, using the CARE approach to communicate and develop policies that provide direction for patient pathways and guidelines in the management of multi-morbid conditions (57). Encounters that promote social prescribing have been touted as an alternative that could reduce polypharmacy, and manage the complexity of multi-morbidity because it is a way of referring frail older adults to healthcare services or to community support groups for practical social support (33). This approach provides a range of unorthodox prescriptive interventions that can be implemented alongside primary care to optimize functioning and reduce unnecessary prescription of medications by involving older persons in participatory activities such as volunteering, music, and exercise to foster togetherness within their social circles.

The use of electronic health records (EHR) is another person-centered approach to reduce the risk associated with polypharmacy. The Electronic Frailty Index (eFI) can be used to foretell adverse events for older patients (58) because it can facilitate a regular comprehensive review of prescriptions from multiple service providers. An accessible electronic medical database would assist physicians in prescribing new drugs, reviewing medication efficacy, and to deprescribe when co-morbid conditions improve (32, 33). This method can potentially prevent avoidable ADR that may result from polypharmacy via drug-drug interactions. Another important aspect of the electronic medical record system is that it contains the results of the administrative and clinical encounters of a patient, therefore EHR interoperability with other provider systems will help prevent extended hospitalization and delayed referrals for older persons due to the compilation of medical history (59). Easy referrals and lack of extended hospitalization reduce the risk of polypharmacy because a reduction in time spent in hospital care reduces further drug prescriptions (60).

Research has shown that patients prioritize empathy and the human aspects of care in clinical and disease management (57). Developing patient pathways from a more patient-centered approach rather than using a disease-specific model in multi-morbid disease management for older patients have been suggested as a way of looking to the future (61). One way to do this is to strengthen effective communication channels between healthcare professionals and older patients to provide opportunities for the patient to express their views and to participate willingly in the medical decision-making process (57). A practical approach to establishing rapport is the self-awareness of healthcare practitioners in how they approach the patient at the bedside, the non-verbal way they introduce themselves, their opening words and sentences as opposed to maintaining less eye contact, talking loudly, raising an eyebrow which are non-verbal communications that may come across as critical (27). Listening more to the patient, summarizing key points of the patient's explanation, revising decisions about the next actions, telling the patient what they are typing in the computer, talking less, signaling with occasional grunts, and helping the patient gain control in situations that require lifestyle changes rather than additional prescription are ways of connecting, accessing and responding to effectively communicate in a helpful and non-patronizing way (27).

The need for effective communication between healthcare providers and older people with multimorbidity is vital for achieving person-centered care and lowering the treatment burden. A report by the NHS (62) outlined that about 70–80% of the population in Scotland with long term conditions are on self-care management due to a lack of effective communication, because nurses in community practice consistently overestimate or underestimate the degree of pain in older adults, and are more likely to perceive them as exaggerating (63). Effective communication between healthcare professionals and the older person with multimorbidity is crucial to gain an understanding of the patient's situation and enable proper diagnosis and administration of treatment.

## Conclusion

Multi-morbidity in older people requires a systematic understanding of its varied complexities in order to inform strategies that equip healthcare practitioners with the right skill-sets to understand the condition and to develop a standardized assessment and treatment tool for providing care for patients with long term conditions. Communication from a person-centered perspective is key to encourage patient interaction and collaboration, and to give clinicians more information that will help them make informed medical decisions to optimize an all-round benefit for the older person with multi-morbidity.

Reducing social and health inequities and redistributing state wealth via direct taxation and conditional cash transfers are important ways countries can quickly mitigate barriers to effectively manage the complexities of multi-morbidity. In addition, the use of electronic health records and the electronic frailty index (EFI) are digital formats that may assist in reducing complications arising from delayed referrals, extended hospitalization, and for providing information on polypharmacy and additional long-term conditions not clearly known. Since polypharmacy is a risk factor for poor health outcomes and mortality, social prescribing can be used as a person-centered alternative intervention to reduce the need for additional drug prescriptions, foster social connectedness, and reduce social exclusion among older people with multi-morbidity.

## Author Contributions

The author confirms being the sole contributor of this work and has approved it for publication.

## Conflict of Interest

The author declares that the research was conducted in the absence of any commercial or financial relationships that could be construed as a potential conflict of interest.
